# MS Amanda 2.0: Advancements in the standalone implementation

**DOI:** 10.1002/rcm.9088

**Published:** 2021-05-05

**Authors:** Viktoria Dorfer, Marina Strobl, Stephan Winkler, Karl Mechtler

**Affiliations:** ^1^ Bioinformatics Research Group University of Applied Sciences Upper Austria Softwarepark 11, 4232 Hagenberg Austria; ^2^ Institute of Molecular Pathology (IMP) Vienna BioCenter (VBC) Campus‐Vienna‐Biocenter 1 Vienna 1030 Austria; ^3^ Institute of Molecular Biotechnology (IMBA) Austrian Academy of Sciences, Vienna BioCenter (VBC) Dr. Bohr‐Gasse 3 Vienna 1030 Austria; ^4^ Gregor Mendel Institute (GMI) Austrian Academy of Sciences, Vienna BioCenter (VBC) Dr. Bohr‐ Gasse 3 Vienna 1030 Austria

## Abstract

**Rationale:**

Database search engines are the preferred method to identify peptides in mass spectrometry data. However, valuable software is in this context not only defined by a powerful algorithm to separate correct from false identifications, but also by constant maintenance and continuous improvements.

**Methods:**

In 2014, we presented our peptide identification algorithm MS Amanda, showing its suitability for identifying peptides in high‐resolution tandem mass spectrometry data and its ability to outperform widely used tools to identify peptides. Since then, we have continuously worked on improvements to enhance its usability and to support new trends and developments in this fast‐growing field, while keeping the original scoring algorithm to assess the quality of a peptide spectrum match unchanged.

**Results:**

We present the outcome of these efforts, MS Amanda 2.0, a faster and more flexible standalone version with the original scoring algorithm. The new implementation has led to a 3–5× speedup, is able to handle new ion types and supports standard data formats. We also show that MS Amanda 2.0 works best when using only the most common ion types in a particular search instead of all possible ion types.

**Conclusions:**

MS Amanda is available free of charge from https://ms.imp.ac.at/index.php?action=msamanda.

## 1 INTRODUCTION

For decades, mass spectrometry has been known as the primary method to analyze proteins in biological samples.^1^, ^2^ A considerable amount of effort has been spent on instruments, technology and also on algorithm development.^3^, ^4^, ^5^, ^6^, ^7^ Different techniques have evolved to identify peptides in mass spectra from bottom‐up mass spectrometry experiments, namely *de novo* identification, database search and spectrum library search. A plethora of different algorithms exist for each analysis category,^8^, ^9^, ^10^ but despite the increasing popularity of spectrum library search in the last years,^11^, ^12^, ^13^ database search is still often the method of choice when it comes to identifying peptides in mass spectra.^14^

In a database search, each spectrum is compared with a list of peptide candidates from a protein database where the peptide mass matches the precursor mass with a certain tolerance. For each peptide candidate a theoretical spectrum, i.e., all potential fragment ions that could occur in a mass spectrum, is calculated, compared with the experimental spectrum and a score is calculated. The peptide candidate with the highest score is then reported.^8^

The score is an essential part of a search engine, one component that distinguishes different algorithms from each other. In a good search engine, the score for each peptide is constructed in a such a way that false identifications can be discriminated from correct identifications, i.e., the higher the score for a peptide spectrum match (PSM), the more likely the PSM is correct.

However, not only a good scoring scheme is essential for a good search engine, but also ease of use and especially maintenance and future development. The scoring scheme of a search engine can be brilliant, but if the code is not maintained and regularly updated to eradicate errors or improve user experience, the algorithm will at some point no longer be used.

In 2014, we published the peptide identification algorithm MS Amanda,^15^ which has been accepted and widely used by the proteomics community.^16^, ^17^, ^18^, ^19^, ^20^, ^21^ Since then, we have worked hard to constantly maintain the software and incorporated user feedback and feature requests, while retaining the original scoring algorithm. In 2018, we released an improved version of MS Amanda available in Thermo Fisher Proteome Discoverer that is able to identify and validate chimeric spectra.^22^

In this paper, we summarize our improvements for the standalone version of MS Amanda, namely:

- Increase in search speed
- Support of multiple spectra and database files
- Support of standardized input and output formats
- Support of common ion types in UVPD spectra
- Improvements in usability

## 2 METHODS

### 2.1 Performance improvements

The first issue we tackled was search speed. In the original version of MS Amanda, it was important to us that the algorithm could run on any machine, independent of the available CPU cores and RAM. Two parameters controlled how many spectra could be processed at once and how many proteins could be searched at the same time, thus defining the speed and – indirectly – the required memory. In addition, already digested protein databases were re‐used in subsequent searches – provided that the digestion parameters, i.e., digestion enzyme type or number of missed cleavages, matched. While this is still true for the new version, we changed the way in which digested FASTA files are stored on the hard disk. In contrast to the first version where we used compressed plain text, we now work with binary encodings. In the first version each protein was digested and its peptides stored individually. Although this allowed for fast database digestion, the subsequent file operations to read the digested peptides were identified as a major bottleneck. We changed this implementation and now peptides with the same sequence are grouped and stored only once. Additional mapping files are generated to keep track of the connection between peptides and proteins. Although the grouping and generation of mapping files takes additional time, the decreased number of files that have to be read still significantly reduces the runtime (see section 3).

While these changes have significantly improved search speed, there was still room for improvement on operating systems other than Windows. For us it was essential that MS Amanda runs on all commonly used operating systems. As MS Amanda is implemented in C#, this was only possible using the mono framework by the time of publication in 2014. While the mono framework was a great way to start, we could still see that the algorithm could not use the full potential of its parallelized implementation on Linux and macOS systems.

In 2016, Microsoft released a new framework, .NET Core, able to run on any operating system. We therefore ported MS Amanda from .NET Framework to .NET Core (which works cross‐platform) to make it available on Windows, macOS and Linux without requiring parallel development. We have tested these performance improvements by using three replicates of HeLa cell lysates measured on a Thermo Fisher QExactive+ (PXD007750, Dataset A^22^).

In addition, users have reported great results achieved using MS Amanda on phosphorylated data sets and it has frequently been used to identify modified peptides.^23^, ^24^, ^25^ We therefore analyzed four phospho‐enriched HeLa cell lysates from the Chorus Project^26^ (https://chorusproject.org/, identifier 1,374, DDA files only) and compared results from MS Amanda 2.0 with a search engine thoroughly accepted by the community: X!Tandem^27^ (version 5.0.1). Analyses were performed using SearchGUI^28^ (version 4.0.18), using a Human Swiss‐Prot database (2020‐12) including common contaminants. Searches were performed using the following parameters: 10 ppm precursor mass tolerance, 0.02 Da fragment mass tolerance, carbamidomethyl (C) as fixed, oxidation (M) and phosphorylation (S,T,Y) as variable modifications, refinement set to false for X!Tandem. All other parameters were left at defaults. Calculation of the false discovery rate (FDR) was performed within PeptideShaker^29^ (version 1.16.13).

In addition, we executed comparative performance tests using the HeLa cell lysates also utilized for the runtime analysis (PXD007750, Dataset A^22^), applying the same parameters and using the same modification settings except for phosphorylation.

### 2.2 Support of multiple spectra and database files

When trying to identify peptides in mass spectra using database search, it is essential to include common contaminants in the list of potential peptide candidates. In the original version of MS Amanda, the algorithm could only handle a single FASTA file. However, these contaminants are normally stored in a separate file, making it necessary to combine the protein database that will be used for the search and the contaminations database prior to starting the search. As this is impractical for users and a possible source of errors, MS Amanda now also accepts a folder containing all FASTA files the spectra should be compared with. The same holds for spectra files. Nowadays, mass spectrometry experiments do not consist of single result files but rather comprise multiple biological and technical replicates or different instrument settings that are compared. We therefore also changed the implementation such that now multiple spectra files can be queued for search at once.

### 2.3 Support of standardized input and output formats

Considerable effort has been put in by the HUPO PSI standardization community to guarantee and enhance communication between tools and algorithms by providing standard data formats for mass spectra and its (peptide) identification results, namely .mzML^30^ and .mzIdentML.^31^, ^32^ We strongly support these efforts as this increases the usability and versatility of algorithms. Providing support for standardized data formats can easily support dissemination of tools and boost utilization of developed tools. We thus enabled MS Amanda to read and write these standard data formats in addition to the file formats supported by the original publication, i.e., .mgf as input file format and .csv as output file format.

### 2.4 Support of common ion types in UVPD spectra

The first version of the MS Amanda algorithm supported ions occurring when using CID,^33^ HCD,^34^ ETD,^35^ and EThcD^36^ fragmentation. A fragmentation technique that has gained increasing attraction in recent years is ultraviolet photodissociation (UVPD).^37^, ^38^ In addition to the common ions such as a, b, x, y, or z fragments, UVPD also often generates additional fragment ions such as a + 1, c, x + 1, or y − 1 ions.^39^, ^40^ Consideration of these ion types for scoring is now also supported by MS Amanda. The Thermo Fisher Proteome Discoverer version of MS Amanda also features these ion types.

### 2.5 Improvements in usability

To enhance usability, we changed the way how to call MS Amanda from the command line by introducing new command line arguments to be able to handle all new features. In addition, the order of input parameters is no longer essential as it was the case for the previous version of MS Amanda. While search parameters are still read from the settings .xml file, parameters such as the file or folder containing spectra, the FASTA file(s) or the desired output format are read as command line parameters (see Table 1 for all available options). Although these named parameters are in contrast to unix command line parameter conventions, where only optional parameters should use option names, we favor this approach due to its higher user‐friendliness. To adhere to the Unix conventions we still support the previous command line call.

**TABLE 1:** All currently available command line parameters for the standalone version. Required parameters are given in bold

Parameter	Description
**‐s**	Spectrum file or spectrum folder (.mgf|.mzML)
**‐d**	Protein|peptide database file or folder (.fasta)
**‐e**	MS Amanda settings file including all search settings (.xml)
‐f	Output file format (1|2), 1: .csv (default), 2: .mzIdentML
‐o	Output file name or folder (default: location of spectrum file)

## 3 RESULTS

### 3.1 Performance comparison

We compared the search speed of the initial implementation of MS Amanda and of the currently available algorithm on all three operating systems. We used three replicates of HeLa cell lysates measured on a Thermo Fisher QExactive+ (see section 2), and compared runtimes of MS Amanda 1.0 and MS Amanda 2.0 on operating systems Windows, Linux and macOS. We used MS Amanda 1.0 (version 1.0.0.4484) and MS Amanda 2.0 (version 2.0.0.17442) on Windows and MS Amanda 1.0 (version 1.0.0.4485) using the mono framework (version 6.12.) and MS Amanda 2.0 (version 2.0.0.17442) on Linux and macOS, running them on systems with the following specifications:

- Windows 10 Pro, Intel® Xeon E3‐1231v3, 3.4 GHz, 8 GB RAM, Samsung SSD 850 EVO
- Ubuntu 20.04, Intel® Xeon E3‐1231v3, 3.4 GHz, 8 GB RAM, Samsung SSD 850 EVO
- macOS Big Sur, v. 11.2.1, Quad‐Core Intel® Core i7, 2.8 GHz, 16 GB RAM, Apple SSD AP1024M

Linux and Windows were set up as virtual machines and benchmarks were performed on the same server, using libvirt version 5.10.0, pinned to 2 cores and 8 GB RAM each. For the first replicate a new digestion of the protein database was generated; the subsequent two replicates re‐used the pre‐digested database.

**FIGURE 1:**
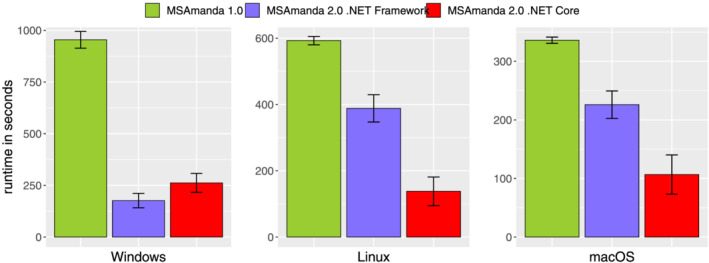
Search speed improvements of MS Amanda 2.0 ported to .NET Core (red) compared with the original implementation MS Amanda 1.0 (green) and MS Amanda 2.0 implemented on the .NET Framework (purple) on the operating systems Windows, Linux and macOS. MS Amanda 1.0 and MS Amanda 2.0.NET Framework were run with the help of mono (version 6.12) on Linux and macOS

Figure 1 shows that, compared with the original version of MS Amanda, MS Amanda 2.0 runs on average more than three times faster on Windows and macOS systems and almost five times faster on Linux. Runtime comparison between operating systems is however only applicable for Linux and Windows, as they were run on the same machine. Data shown in Figure 1 are average runtimes of each replicate including the database digestion for the first file, i.e., a comparison of a fresh installation of both MS Amanda versions. On average, database digestion accounted for 8% of the total runtime for the old version (60, 63, and 13 seconds on Windows, Linux and Mac, respectively) but increased to on average 42% of the total runtime for the latest version (95, 97, 73 seconds for Windows, Linux, and Mac, respectively). Still, the performance gain through the adapted digestion handling outweighed these losses.

We also investigated the impact of porting MS Amanda from the .NET Framework to .NET Core, which makes the mono framework obsolete. We compared runtimes of the last version of MS Amanda prior to the .NET version change (MS Amanda 2.0 vs 2.0.0.14828) to the current version of MS Amanda. While this leads to a slight increase in runtime on the Windows operating system, the .NET Core versions running on Linux and macOS were 2 to 2.5 times faster than those versions using the mono framework. This fact and the advantage of just a single implementation for all operating systems further convinces us that .NET Core was a good choice.

When comparing the number of identified PSMs at 1% FDR of non‐phosphorylated and phosphorylated HeLa data sets, we found that for the non‐phosphorylated HeLa data set results from MS Amanda and X!Tandem are comparable (12,092 vs 12,179 PSMs at 1% FDR). However, MS Amanda outperforms X!Tandem on the phosphorylated data, as MS Amanda is able to identify on average 13% more PSMs at 1% FDR as compared with X!Tandem (13,175 vs 11,704 PSMs at 1% FDR).

### 3.2 Identification results for UVPD spectra

**FIGURE 2:**
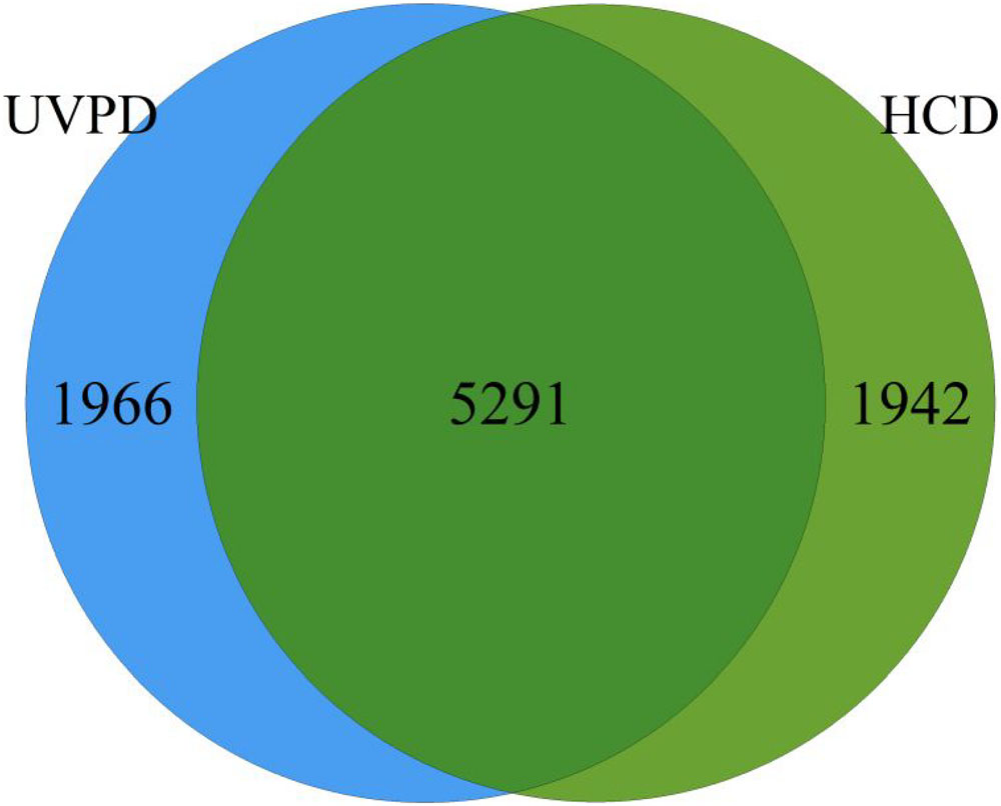
Overlap of unique peptides at 1% FDR for UVPD and HCD results of a single replicate

Several groups have reported the common occurrence of a + 1, x + 1, and y − 1 ions in UVPD spectra.^39^, ^40^ We wanted to investigate the applicability of these ion types to be used for scoring and tested various ion settings on HeLa samples measured on a Thermo Fisher QExactive using UVPD peptide fragmentation (PXD003109^39^). In their manuscript, Fort and co‐workers^39^ compared UVPD and HCD fragmentation techniques and claimed that both techniques generated a comparable number of reliable identifications. Our results support these findings. In addition, the overlap of identified unique peptides between these two techniques matches the outcome of Fort and colleagues^39^ (see Figure 2). However, we see that the identification quality strongly depends on the ion types considered to compare peptides to spectra. As we have seen during our research of the original MS Amanda publication, the MS Amanda algorithm works best when the most frequently seen ion types are used for scoring, in contrast to all potential ion types that might occur. For HCD, e.g., the highest number of identifications can be achieved when using b and y ions only. This is due to the probability score applied in MS Amanda. The more ion types are considered the more potential ion candidates are available – this holds also for random peptides that may lead to false identifications – and therefore the higher the probability to match random peaks by chance.

**FIGURE 3:**
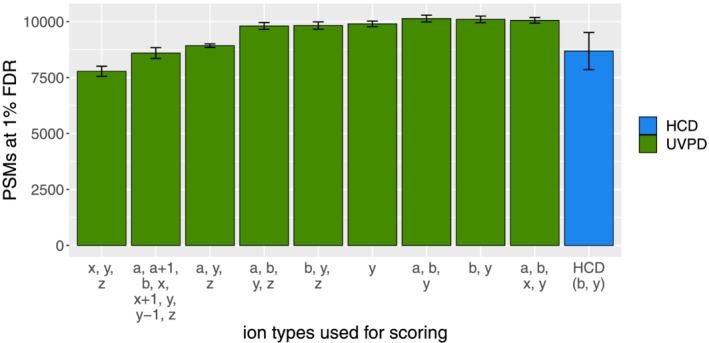
Impact of rare ion types: Considering ion types in the score that are rather rare has a huge impact on identification results

**FIGURE 4:**
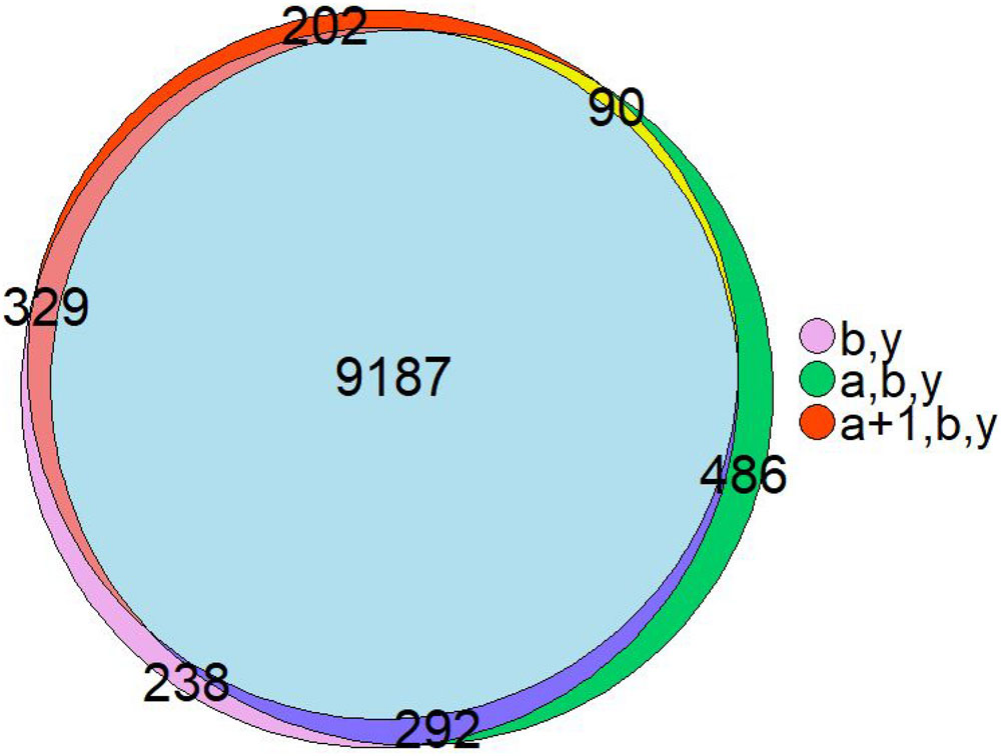
Overlap of PSMs at 1% FDR for different ion type settings when searching UVPD spectra. Including or excluding a/a + 1 ions has no significant impact on the search results

For UVPD spectra, we see a similar effect. Despite the fact that x + 1, a + 1, and y − 1 ions occur regularly in these spectra, they are still less common than a, b, or y ions. As depicted in Figure 3, using all these ion types that might occur in UVPD spectra decreases the number of identified PSMs at 1% FDR by 15%. Leaving out common ion types, however, is even worse, as this yields 23% less identifications. Therefore, for MS Amanda 2.0 it is best to search only for the most common ion types also in UVPD spectra. We assume this might be similar for other search engines using probability‐based scores. In addition, we also compared the identified PSMs at 1% FDR when a and a + 1 ions were included or excluded as ion type. The comparison has been made on a spectrum‐by‐spectrum basis as proposed by Agten and co‐workers.^41^ Figure 4 reveals that the difference in identifications for these settings is negligible, indicating that solely b and y ions could be used here as ion types in the search.

## 4 CONCLUSIONS

Valuable software in general is not only defined by powerful algorithms but also by continuous maintenance and development. This is of course also true for mass spectrometry software. Several years ago, we published our peptide identification algorithm MS Amanda, showing that we are able to outperform algorithms very frequently used by the community. In this manuscript we show that the MS Amanda implementation has advanced, reacting to user needs and feature requests. The latest standalone version of MS Amanda, MS Amanda 2.0, has numerous improvements compared with the first published version, including increase in search speed, support of multiple FASTA and spectra files, support of standardized formats (.mzML and .mzIdentML), new ion types occurring in UVPD spectra, and usability improvements. In addition, MS Amanda 2.0 has been ported from the .NET Framework to .NET Core 3.1, being able to run on all operation systems without the usage of the mono framework. With that change and other improvements, we have shown that the latest version of MS Amanda is now 3.2–4.3 times faster than the initial version, depending on the operating system used. MS Amanda is now even more flexible and widely applicable to all sorts of mass spectrometry data. The standalone version of MS Amanda can also be used within SearchGUI^28^ and results can be analyzed using PeptideShaker.^29^

Of course, the further development of MS Amanda is an ongoing endeavor. We are currently working on supporting chimeric spectra identification published as the CharmeRT workflow also in the standalone version. In addition, we are working on an automated pin file generation to be able to validate MS Amanda results with Percolator.^42^
